# *KCNJ10* May Not Be a Contributor to Nonsyndromic Enlargement of Vestibular Aqueduct (NSEVA) in Chinese Subjects

**DOI:** 10.1371/journal.pone.0108134

**Published:** 2014-11-05

**Authors:** Jiandong Zhao, Yongyi Yuan, Shasha Huang, Bangqing Huang, Jing Cheng, Dongyang Kang, Guojian Wang, Dongyi Han, Pu Dai

**Affiliations:** 1 Department of Otolaryngology, PLA General Hospital, Beijing, People’s Republic of China; 2 Department of Otolaryngology, Hainan Branch of PLA General Hospital, Sanya, People’s Republic of China; NIDCR/NIH, United States of America

## Abstract

**Background:**

Nonsyndromic enlargement of vestibular aqueduct (NSEVA) is an autosomal recessive hearing loss disorder that is associated with mutations in *SLC26A4*. However, not all patients with NSEVA carry biallelic mutations in *SLC26A4*. A recent study proposed that single mutations in both *SLC26A4* and *KCNJ10* lead to digenic NSEVA. We examined whether *KCNJ10* excert a role in the pathogenesis of NSEVA in Chinese patients.

**Methods:**

*SLC26A4* was sequenced in 1056 Chinese patients with NSEVA. *KCNJ10* was screened in 131 patients who lacked mutations in either one or both alleles of *SLC26A4.* Additionally, *KCNJ10* was screened in 840 controls, including 563 patients diagnosed with NSEVA who carried biallelic *SLC26A4* mutations, 48 patients with nonsyndromic hearing loss due to inner ear malformations that did not involve enlargement of the vestibular aqueduct (EVA), 96 patients with conductive hearing loss due to various causes, and 133 normal-hearing individuals with no family history of hereditary hearing loss.

**Results:**

925 NSEVA patients were found carrying two-allele pathogenic *SLC26A4* mutations. The most frequently detected *KCNJ10* mutation was c.812G>A (p.R271H). Compared with the normal-hearing control subjects, the occurrence rate of c.812G>A in NSEVA patients with lacking mutations in one or both alleles of *SLC26A4* had no significant difference(1.53% vs. 5.30%, χ^2^ = 2.798, p = 0.172), which suggested that it is probably a nonpathogenic benign variant. *KCNJ10* c.1042C>T (p.R348C), the reported EVA-related mutation, was not found in patients with NSEVA who lacked mutations in either one or both alleles of *SLC26A4.* Furthermore, the normal-hearing parents of patients with NSEVA having two *SLC26A4* mutations carried the *KCNJ10* c.1042C>T or c.812G>A mutation and a *SLC26A4* pathogenic mutation.

**Conclusion:**

*SLC26A4* is the major genetic cause in Chinese NSEVA patients, accounting for 87.59%. *KCNJ10* may not be a contributor to NSEVA in Chinese population. Other genetic or environmental factors are possibly play a role in the etiology of Chinese EVA patients with zero or monoallelic *SLC26A4* mutation.

## Introduction

Nonsyndromic sensorineural hearing loss associated with an enlargement of the vestibular aqueduct (EVA) is a common form of inner ear malformation [1]. It occurs either congenitally or following some mild head injury and results in fluctuating and progressive hearing loss. Nonsyndromic enlargement of vestibular aqueduct (NSEVA) is an autosomal recessive hearing loss associated with mutations in the anion transporter *SLC26A4*, which encodes the anion transporter protein pendrin [2]. Located in the kidney, thyroid, and inner ear, pendrin is responsible for the transport of Cl^–^, HCO3^–^, OH^–^, I^–^, and formate [2], [3]. In the inner ear, pendrin is expressed mainly in the external sulcus, endolymphatic duct and sac, utricle, and saccule, and it maintains the endolymphatic balance by mediating Cl^−^/HCO_3_^–^ exchange [4]–[6]. Malfunction of the *SLC26A4* protein can cause NSEVA and Pendred syndrome (PS), a type of syndromic sensorineural hearing loss characterized by EVA, goiter, and in some cases Mondini malformation [7].

The underlying mechanisms for the etiology of NSEVA/PS remain elusive; however, studies that have screened for *SLC26A4* mutations in patients with NSEVA/PS and subsequent studies of these mutant proteins have been informative [2]. Some pendrin mutants fail to reach the cell surface and are retained in the endoplasmic reticulum, impairing anion transport and consequently the ionic balance of endolymph [8], [9]. This imbalance causes endolymphatic dilatation, enlarges the membranous labyrinth, and disrupts the development of bone structures in the inner ear [9]. In addition, studies using *Slc26a4* ^−/−^ mouse models have demonstrated that the loss of pendrin expression decreases HCO_3_^–^ secretion into the endolymph [10]. The resulting endolymphatic acidification inhibits Ca^2+^ reabsorption from endolymph, which further inhibits sensory transduction and promotes the degeneration of the sensory hair cells [10]. In China, NSEVA accounts for 20–25% of hereditary hearing loss cases, and biallelic mutations in *SLC26A4* represent nearly 90% of the genetic etiology of NSEVA [11]. However, the detection rate of *SLC26A4* biallelic mutations varies among different ethnicities (*e.g.*, 24% in Caucasians and 81% in Koreans) [12], [13].

Although most previous studies in patients with NSEVA have focused on *SLC26A4*, not all patients with NSEVA carry biallelic mutations in *SLC26A4*. This discrepancy has led to the hypothesis that other genetic modifications or environmental factors may be involved. Yang et al. [14] proposed that single mutations in both *SLC26A4* and *KCNJ10* lead to digenic NSEVA. *KCNJ10* encodes a 42.5-kD member of the inward rectifier potassium channel family and is expressed mainly in the brain, kidney, and inner ear. The *KCNJ10* K^+^ channel buffers the K^+^ levels in brain glial cells, and mutations in this gene may lead to the so-called ‘EAST/SeSAME syndrome,’ which is manifested by seizures, sensorineural deafness, ataxia, mental retardation, and electrolyte imbalance [15], [16]. In the inner ear, *KCNJ10* is expressed mainly in the intermediate cells of the stria vascularis. It maintains the K^+^ concentration in the endolymph and generates endocochlear potential, which is the main driving force of sensory transduction that enables hearing [17], [18]. In *Kcnj10* ^−/−^ mice, the endocochlear potential is abolished and hearing is greatly compromised [18].

Interestingly, studies using *Slc26a4* ^−/−^ mice have revealed an association between *SLC26A4* and *KCNJ10.* In these studies, the loss of pendrin reduced the protein expression of *KCNJ10*, which in turn eliminated endocochlear potential and impaired hearing [10], [19]. Furthermore, Yang and colleagues have identified patients with NSEVA who carried single mutations in both *SLC26A4* and *KCNJ10* genes [14]. In one affected patient, Yang and colleagues identified the *KCNJ10* c.1042C>T (p.R348C) mutation as being in double heterozygosity with a *SLC26A4* mutation, whereas this was not observed in controls of European or Chinese descent with normal hearing [14]. In addition, they showed that haplo-insufficiency of *SLC26A4* in *Slc26a4* ^+/−^ mice reduced the *KCNJ10* levels. The *KCNJ10* mutants identified in their study displayed reduced K^+^ conductance activity, a critical step in generating endocochlear potential [14]. Furthermore, the *KCNJ10* c.1042C>T mutation, which was suggested to be a potentially pathological mutation, was shown to reduce K^+^ conductance activity [14]. Based on these findings, the authors postulated that single mutations in both *SLC26A4* and *KCNJ10* lead to digenic NSEVA.

In this study, we aimed to examine whether *KCNJ10* plays a role in the pathogenesis of NSEVA by screening *KCNJ10* mutations in Chinese patients with NSEVA who lacked mutations in one or both alleles of *SLC26A4*. This screening resulted in the identification of common *KCNJ10* mutations in the Chinese population. The conclusions from this study contribute important foundations for genetic diagnosis as well as prenatal diagnosis of deafness.

## Subjects and Methods

### Subjects

The subjects were recruited by the Department of Otolaryngology and Genetic Testing Center for Deafness, PLA General Hospital, BeiJing, China. Totally 1056 EVA patients were enrolled in the study. They were all screened the coding region of *SLC26A4* firstly. Then a cohort of 131 Chinese patients with NSEVA without mutation or with only one *SLC26A4* mutation was recruited for mutation screening for *KCNJ10*. In addition, we examined 840 controls, comprising the following four groups: 563 patients who were diagnosed with NSEVA and carried biallelic *SLC26A4* mutations, 48 patients with nonsyndromic hearing loss and inner ear malformation other than EVA, 96 patients with conductive hearing loss due to various causes (70 cases with chronic otitis media, 11 cases with secretory otitis media, 9 cases with conductive deafness due to unknown causes, three cases with otosclerosis, 2 cases with external auditory canal tumor, and 1 case with a jugular tumor sphere), and 133 normal-hearing individuals with no family history of hereditary hearing loss. Written informed consent was obtained from all subjects or guardians prior to blood sampling and genetic testing. The study protocol, including the consent procedure, was performed with the approval of the Ethics Committee of the Chinese PLA General Hospital.

### Mutation screening of *KCNJ10*

Genomic DNA was isolated from whole blood through a modification of standard procedures [20]. Most DNA samples were collected by and stored at the Genetic Testing Center for Deafness. The only coding exon of *KCNJ10* was amplified in a 20-µl PCR reaction mixture containing 20 ng of genomic DNA template, 0.0625 units of Taq DNA polymerase (Biomed, Cat: Pc01, Lot: 211841XB), and 40 nM primers. The following three sets of PCR primers were used (5′→3′): forward, CGATAACCTCCATTATGCTG and reverse, AGGATGGTGGTGAGCACCAG; forward, TGGCTTCCGCTACATCAGTG and reverse, ACAACTTGGTCAAAAAGGCTAA; forward, ACCCCTTACCTTCTATCATG and reverse, GTAGTATTCCTTACCAGGGC. To increase the specificity and sensitivity of PCR amplification, the technique of touchdown-PCR was employed: one cycle at 95°C for 1 min; 95°C for 30 s, 62°C for 30 s, and 72°C for 45 s, followed by 13 cycles at decreasing annealing temperatures in decrements of 0.5°C; 21 cycles of 95°C for 30 s, 56°C for 30 s, and 72°C for 45 s, with a final extension at 72°C for 7 min. The sizes of the PCR products were confirmed by 1% agarose gel electrophoresis. PCR products were sequenced using forward primers to screen for mutations in *KCNJ10* (Doobio Biotechnology Co., China). The detected mutations were confirmed by sequencing using reverse primers.

### Statistics

SPSS 17.0 software was used in statistical analysis. Comparisons between groups were tested using the χ^2^ or Fisher’s exact tests. P value<0.05 was set for statistically significant difference.

## Results

### *SLC26A4* analysis in NSEVA patients

Totally 925 NSEVA patients were found carrying two-allele pathogenic *SLC26A4* mutations, *SLC26A4* accounts for 87.59% (925/1056) of the genetic etiology in Chinese NSEVA patients. The *SLC26A4* mutation data were shown in Table 1 and Table 2.

**Table 1:** Genotype of NSEVA patients with two-allele *SLC26A4* mutations.

Number	SLC26A4 Genotype		Number of patients
	Allele 1	Allele 2	
1	IVS7-2A>G	IVS7-2A>G	237
2	IVS7-2A>G	c.2168A>G(p.H723R)	103
3	IVS7-2A>G	c.1226G>A(p.R409H)	40
4	IVS7-2A>G	c.1975G>C(p.V659L)	35
5	IVS7-2A>G	c.1229C>T(p.T410M)	27
6	IVS7-2A>G	c.1174A>T(p.N392Y)	23
7	IVS7-2A>G	c.2027T>A(p.L676Q)	19
8	IVS7-2A>G	c.589G>A(p.G197R)	15
9	IVS7-2A>G	IVS15+5G>A	11
10	IVS7-2A>G	c.917insG	10
11	c.2168A>G(p.H723R)	c.2168A>G(p.H723R)	9
12	c.2168A>G(p.H723R)	c.1975G>C(p.V659L)	9
13	c.2168A>G(p.H723R)	c.1229C>T(pT410M)	8
14	IVS7-2A>G	c.281C>T(p.T94I)	6
15	IVS7-2A>G	c.1336C>T(p.Q446X)	6
16	IVS7-2A>G	c.1262A>C(p.Q421P)	5
17	IVS7-2A>G	IVS4+2T>C	5
18	c.1174A>T(p.N392Y)	c.1226G>A(p.R409H)	5
19	IVS7-2A>G	c.1225C>T(p.R409C)	5
20	IVS7-2A>G	c.1586T>G(p.I529S)	5
21	IVS7-2A>G	c.1687_1692insA	5
22	IVS7-2A>G	c.235C>T(p.R79X)	4
23	c.1229C>T(p.T410M)	c.1229C>T(p.T410M)	4
24	c.1975G>C(p.V659L)	c.2027T>A(p.L676Q)	4
25	IVS7-2A>G	c.1079C>T(p.A360V)	4
26	IVS7-2A>G	IVS13+9C>T	4
27	c.2168A>G(p.H723R)	c.2027T>A(p.L676Q)	4
28	c.2168A>G(p.H723R)	c.1226G>A(p.R409H)	4
29	IVS7-2A>G	c.1318A>T(p.K440X)	4
30	c.1540C>T(p.Q514X)	c.2168A>G(p.H723R)	3
31	c.2027T>A(p.L676Q)	c.589G>A(p.G197R)	3
32	IVS7-2A>G	c.1340delA	3
33	IVS7-2A>G	c.563T>C(p.I188T)	3
34	IVS7-2A>G	c.170C>A(p.S57X)	3
35	IVS7-2A>G	c.1594A>C(p.S532R)	3
36	IVS7-2A>G	c.946G>T(p.G316X)	3
37	IVS7-2A>G	c.1334T>G(p.L445W)	3
38	IVS7-2A>G	c.1343C>A(p.S448X)	3
39	c.1975G>C(p.V659L)	c.1229C>T(p.T410M)	3
40	IVS7-2A>G	c.2162C>T(p.T721M)	3
41	IVS7-2A>G	c.1540C>T(p.Q514X)	3
42	IVS7-2A>G	c.1693insA	3
43	IVS7-2A>G	c.1160C>T(p.A387V)	3
44	IVS7-2A>G	c.2167C>G(p.H723D)	3
45	IVS7-2A>G	c.1343C>A(p.S448X)	3
46	c.2168A>G(p.H723R)	c.563T>C(p.I188T)	3
47	IVS7-2A>G	c.1520delT	3
48	c.2168A>G(p.H723R)	c.589G>A(p.G197R)	3
49	IVS7-2A>G	c.1173C>A(p.S391R)	3
50	c.1229C>T(p.T410M)	c.1174A>T(p.N392Y)	2
51	IVS7-2A>G	c.414delT	2
52	IVS7-2A>G	c.1548insC	2
53	c.1174A>T(p.N392Y)	IVS15+5G>A	2
54	IVS7-2A>G	IVS14-2A>G	2
55	c.235C>T(p.R79X)	c.2168A>G(p.H723R)	2
56	IVS7-2A>G	c.1699A>T(p.K567X)	2
57	IVS7-2A>G	c.1371C>A(p.N457K)	2
58	1240–1243GAGA>AAAG(p.E414K, p.S415G)	IVS14-6T>G	2
59	IVS7-2A>G	c.1670G>A(p.G557D)	2
60	IVS7-2A>G	c.665G>T(p.G222V)	2
61	IVS7-2A>G	c.1990G>A(p.A664T)	2
62	IVS7-2A>G	c.1238A>G(p.Q413R)	2
63	c.1079C>T(p.A360V)	c.2168A>G(p.H723R)	2
64	c.1229C>T(p.T410M)	c.1687_1692insA	2
65	IVS7-2A>G	c.1022delC	2
66	IVS7-2A>G	c.907G>C(p.E303Q)	2
67	IVS7-2A>G	c.2086C>T(p.Q696X)	2
68	c.2027T>A(p.L676Q)	c.2027T>A(p.L676Q)	2
69	IVS7-2A>G	c.269C>T(p.S90L)	2
70	c.1174A>T(p.N392Y)	c.2168A>G(p.H723R)	2
71	IVS7-2A>G	c.1363A>T(p.I455F)	2
72	c.1174A>T(p.N392Y)	c.2027T>A(p.L676Q)	2
73	c.1673A>T(p.N558I)	IVS14+1G>A	2
74	c.1318A>T(p.K440X)	c.1327G>C(p.E443Q)	2
75	IVS7-2A>G	c.1991C>T(p.A664V)	2
76	IVS7-2A>G	c.1595G>T(p.S532I)	2
77	c.1226G>A(p.R409H)	c.1226G>A(p.R409H)	2
78	c.946G>T(p.G316X)	c.2168A>G(p.H723R)	2
79	IVS7-2A>G	c.668T>C(p.F223S)	2
80	IVS7-2A>G	c.1970G>T(p.S657I)	2
81	c.1522A>G(p.T508A)	c.1522A>G(p.T508A)	1
82	c.1595G>T(p.S532I)	IVS4+7A>G	1
83	IVS7-2A>G	c.1720G>A(p.A574T)	1
84	c.1343C>A(p.S448X)	c.2167C>G(p.H723D)	1
85	c.1178delTCT	c.439A>G((p.M147V)	1
86	IVS7-2A>G	c.400A>G(p.R134G)	1
87	c.589G>A(p.G197R)	c.1105A>G(p.K369E)	1
88	IVS15+5G>A	c.1318A>T(p.K440X)	1
89	IVS7-2A>G	c.1367C>A(p.A456D)	1
90	IVS7-2A>G	c.249G>A(p.W83X)	1
91	c.1174A>T(p.N392Y)	c.1975G>C(p.V659L)	1
92	IVS7-2A>G	c.1964T>A(p.I655N)	1
93	IVS7-2A>G	c.2081delC	1
94	c.281C>T(p.T94I)	c.692T>A(p.V231E)	1
95	IVS7-2A>G	IVS9+1G>A	1
96	c.2168A>G(p.H723R)	c.439A>G((p.M147V)	1
97	IVS7-2A>G	IVS10+1G>A	1
98	c.890delC	IVS7-2A>G	1
99	IVS7-2A>G	c.1829C>A(p.S610X)	1
100	IVS7-2A>G	c.218delA	1
101	c.1594A>C(p.S532R)	c.2168A>G(p.H723R)	1
102	c.1229C>T(p.T410M)	c.281C>T(p.T94I)	1
103	IVS7-2A>G	c.404A>G(p.H135R)	1
104	c.1022insC	c.1489G>A(p.G497S)	1
105	c.946G>T(p.G316X)	c.1343C>A(p.S448X)	1
106	IVS7-2A>G	c.230A>T(p.K77I)	1
107	IVS7-2A>G	c.1327G>C(p.E443Q)	1
108	c.1226G>A(p.R409H)	c.58T>C(p.Y20H)	1
109	c.2027T>A(p.L676Q)	IVS13+9C>T	1
110	c.249G>A(p.W83X)	c.2168A>G(p.H723R)	1
111	c.203T>C(p.L68P)	IVS4+2T>C	1
112	c.911T>A(p.V304E)	c.1238A>G(p.Q413R)	1
113	c.2027T>A(p.L676Q)	IVS15+5G>A	1
114	c.1343C>A(p.S448X)	c.1339delA	1
115	c.1976T>G(p.V659G)	c.2168A>G(p.H723R)	1
116	c.2027T>A(p.L676Q)	c.1520delT	1
117	IVS10+1G>A	IVS10+1G>A	1
118	c.1238A>G(p.Q413R)	c.1172G>A(p.S391N)	1
119	IVS7-2A>G	c.1634T>A(p.V545E)	1
120	IVS7-2A>G	c.2179C>T(p.L727F)	1
121	c.1343C>A(p.S448X)	c.1178delTCT	1
122	c.2086C>T(p.Q696X)	c.2168A>G(p.H723R)	1
123	c.754T>C(p.S252P)	c.86A>G(p.E29G)	1
124	c.2168A>G(p.H723R)	IVS4+2T>C	1
125	c.1229C>T(p.T410M)	c.1949T>A(p.V650D)	1
126	IVS7-2A>G	IVS5+2T>A	1
127	c.1229C>T(p.T410M)	c.1693insA	1
128	c.391G>A(p.G131R)	c.2168A>G(p.H723R)	1
129	IVS7-2A>G	c.2014G>A(p.G672R)	1
130	IVS7-2A>G	IVS12+1G>A	1
131	IVS7-2A>G	c.1373T>C(p.L458P)	1
132	c.754T>C(p.S252P)	c.2168A>G(p.H723R)	1
133	c.71G>A(p.R24Q)	c.2168A>G(p.H723R)	1
134	c.1174A>T(p.N392Y)	c.1829C>A(p.S610X)	1
135	c.439A>G((p.M147V)	c.1615A>G(p.I539V)	1
136	IVS7-2A>G	c.1615A>G(p.I539V)	1
137	c.1174A>T(p.N392Y)	c.1340delA	1
138	c.1975G>C(p.V659L)	c.1238A>G(p.Q413R)	1
139	c.589G>A(p.G197R)	c.589G>A(p.G197R)	1
140	c.1174A>T(p.N392Y)	c.249G>A(p.W83X)	1
141	c.1991C>T(p.A664V)	c.1336C>T(p.Q446X)	1
142	IVS7-2A>G	c.349delC	1
143	IVS7-2A>G	c.259G>T(p.D87Y)	1
144	IVS7-2A>G	c.1687_1692delA	1
145	c.109G>T(p.E37X)	c.2168A>G(p.H723R)	1
146	c.917insG	c.2168A>G(p.H723R)	1
147	c.1226G>A(p.R409H)	IVS15+5G>A	1
148	IVS7+1G>A	c.2168A>G(p.H723R)	1
149	c.1334T>G(p.L445W)	c.1544T>C(p.F515S)	1
150	c.1489G>A(p.G497S)	c.414delT	1
151	IVS7-2A>G	c.1803G>C(p.K601N)	1
152	c.2168A>G(p.H723R)	c.279T>A(p.S93N)	1
153	c.235C>T(p.R79X)	c.589G>A(p.G197R)	1
154	IVS15+5G>A	c.2145G>T(p.K715N)	1
155	c.1229C>T(p.T410M)	c.1343C>A(p.S448X)	1
156	IVS7-2A>G	c.79T>A(p.Y27N)	1
157	c.1586T>G(p.I529S)	c.2168A>G(p.H723R)	1
158	IVS7-2A>G	c.2009T>C(p.V670A)	1
159	c.2027T>A(p.L676Q)	c.1586T>G(p.I529S)	1
160	c.1229C>T(p.T410M)	IVS19+2T>A	1
161	c.1264G>A(p.V422I)	c.2027T>A(p.L676Q)	1
162	IVS7-2A>G	c.1975G>C(p.V659L)	1
163	c.109G>T(p.E37X)	c.109G>T(p.E37X)	1
164	c.421T>C(p.F141L)	c.1343C>A(p.S448X)	1
165	IVS7-2A>G	c.227C>T(p.P76L)	1
166	c.1520delT	c.2168A>G(p.H723R)	1
167	c.249G>A(p.W83X)	c.1105A>G(p.K369E)	1
168	IVS7-2A>G	c.754T>C(p.S252P)	1
169	c.279T>A(p.S93N)	c.2183insT	1
170	c.1991C>T(p.A664V)	c.1173C>A(p.S391R)	1
171	c.1343C>A(p.S448X)	c.2168A>G(p.H723R)	1
172	c.249G>A(p.W83X)	c.1340delA	1
173	IVS7-2A>G	c.2168A>C(p.H723P)	1
174	c.1174A>T(p.N392Y)	c.1991C>T(p.A664V)	1
175	c.1975G>C(p.V659L)	c.2039delT	1
176	c.1975G>C(p.V659L)	c.281C>T(p.T94I)	1
177	IVS7-2A>G	c.1714T>G(p.F572V)	1
178	IVS7-2A>G	c.2000T>C(p.F667S)	1
179	c.574delC	c.2168A>G(p.H723R)	1
180	IVS7-2A>G	c.1835delA	1
181	c.1229C>T(p.T410M)	c.1079C>T(p.A360V)	1
182	c.1229C>T(p.T410M)	IVS1-2A>C	1
183	c.1334T>G(p.L445W)	c.2168A>G(p.H723R)	1
184	IVS7-2A>G	c.439A>G((p.M147V)	1
185	c.1318A>T(p.K440X)	c.2168A>G(p.H723R)	1
186	c.1229C>T(p.T410M)	c.1226G>A(p.R409H)	1
187	c.1517T>G(p.L506R)	c.2162C>T(p.T721M)	1
188	c.1174A>T(p.N392Y)	IVS13+9C>T	1
189	c.1174A>T(p.N392Y)	c.1334T>G(p.L445W)	1
190	c.917insG	c.281C>T(p.T94I)	1
191	c.1174A>T(p.N392Y)	c.668T>C(p.F223S)	1
192	c.1229C>T(p.T410M)	c.1594AG>T	1
193	c.2027T>A(p.L676Q)	c.334C>T(p.P112S)	1
194	IVS15+5G>A	c.1520delT	1
195	c.387delC	c.2168A>G(p.H723R)	1
196	c.946G>T(p.G316X)	c.1522A>G(p.T508A)	1
197	IVS7-2A>G	c.587T>A(p.V196D)	1
198	IVS7-2A>G	c.1927G>T(p.E643X)	1
199	c.1687_1692insA	c.68C>A(p.S23X)	1
200	c.1829C>A(p.S610X)	c.2168A>G(p.H723R)	1
201	c.1343C>T(p.S448L)	c.2168A>G(p.H723R)	1
202	c.1174A>T(p.N392Y)	c.414delT	1
203	IVS7-2A>G	c.2118C>A(p.C706X)	1
204	c.2168A>G(p.H723R)	c.281C>T(p.T94I)	1
205	IVS7-2A>G	c.496delA	1
206	c.1540C>T(p.Q514X)	IVS13+9C>T	1
207	c.917insG	c.1229C>T(p.T410M)	1
208	c.2168A>G(p.H723R)	c.1548insC	1
209	IVS7-2A>G	c.1286C>A(p.A429E)	1
210	IVS15+5G>A	c.235C>T(p.R79X)	1
211	c.946G>T(p.G316X)	c.1226G>A(p.R409H)	1
212	c.1927G>T(p.E643X)	c.2168A>G(p.H723R)	1
213	c.2027T>A(p.L676Q)	c.1336C>T(p.Q446X)	1
214	c.1336C>T(p.Q446X)	c.1520delT	1
215	c.2T>C(p.M1T)	c.2168A>G(p.H723R)	1
216	c.1174A>T(p.N392Y)	c.1225C>T(p.R409C)	1
217	c.1174A>T(p.N392Y)	c.346G>A(p.G116S)	1
218	IVS7-2A>G	c.398C>T(p.S133L)	1
219	c.1226G>A(p.R409H)	c.281C>T(p.T94I)	1
220	c.917insG	c.2086C>T(p.Q696X)	1
221	IVS7-2A>G	c.1216G>A(p.A406T)	1
222	c.946G>T(p.G316X)	c.1586T>G(p.I529S)	1
223	c.1173C>A(p.S391R)	c.279T>A(p.S93N)	1
224	c.1985G>A(p.C662Y)	c.1687_1692insA	1
225	c.227C>T(p.P76L)	IVS4+2T>C	1
226	c.768delG	IVS15+5G>A	1
227	c.1174A>T(p.N392Y)	c.281C>T(p.T94I)	1
228	c.1225C>T(p.R409C)	c.1226G>A(p.R409H)	1
229	IVS9+1G>A	IVS18+3A>C	1
230	IVS7-2A>G	c.149T>C(p.L50P)	1
231	c.1174A>T(p.N392Y)	c.1594A>C(p.S532R)	1
232	IVS7-2A>G	c.1339delA	1
233	c.281C>T(p.T94I)	c.1318A>T(p.K440X)	1
234	c.1985G>A(p.C662Y)	IVS7-2A>G	1
235	c.1548insC	c.2086C>T(p.Q696X)	1
236	c.235C>T(p.R79X)	c.1022delC	1
237	c.1226G>A(p.R409H)	IVS5+2T>A	1
238	c.1229C>T(p.T410M)	c.279T>A(p.S93N)	1
239	IVS7-2A>G	c.1746delG	1
240	c.1226G>A(p.R409H)	c.589G>A(p.G197R)	1
241	c.920C>A(p.T307K)	c.2174insCTAT	1
242	IVS7-2A>G	c.1264G>A(p.V422I)	1
243	c.1229C>T(p.T410M)	c.365delT	1
244	IVS7-2A>G	IVS7+2T>C	1
245	c.1229C>T(p.T410M)	c.235C>T(p.R79X)	1
246	c.1174A>T(p.N392Y)	c.1174A>T(p.N392Y)	1
247	c.754T>C(p.S252P)	c.349insC	1
248	IVS7-2A>G	c.387delC	1
249	c.2014G>A(p.G672R)	c.2168A>G(p.H723R)	1
250	c.249G>A(p.W83X)	c.281C>T(p.T94I)	1
251	c.1262A>C(p.Q421P)	c.2054G>T(p.R685I)	1
252	c.1687_1692insA	c.589G>A(p.G197R)	1
253	IVS7-2A>G	c.716T>A(p.V239D)	1
254	c.1229C>T(p.T410M)	c.1546C>T(p.P516S)	1
255	IVS7-3C>G	IVS15+5G>A	1
256	c.1975G>C(p.V659L)	c.2086C>T(p.Q696X)	1
257	c.1225C>T(p.R409C)	c.1225C>T(p.R409C)	1
258	c.2168A>G(p.H723R)	c.404A>G(p.H135R)	1
259	IVS7-2A>G	IVS3+84G>A	1
260	c.2006A>T(p.D669V)	c.439A>G((p.M147V)	1
261	IVS7-2A>G	c.403C>T(p.H135Y)	1
262	IVS7-2A>G	c.1673A>T(p.N558I)	1
263	c.1226G>A(p.R409H)	c.1340delA	1

**Table 2:** Genotype of NSEVA patients with zero or one allele *SLC26A4* mutation.

Number	Genotype	Number of patients
1	IVS7-2A>G/wt	68
2	c.2168 A>G(p.H723R)/wt	11
3	c.2027T>A(p.L676Q)/wt	5
4	c.1174A>T(p.N392Y)/wt	5
5	IVS15+5G>A/wt	4
6	c.1229C>T(p.T410M)/wt	4
7	c.1226G>A(p.R409H)/wt	3
8	c.147C>G(p.S49R)/wt	2
9	c.235C>T(p.R79X)/wt	1
10	c.487G>C(p.R163L)/wt	1
11	c.471C>T(p.P157P)/wt	1
12	c.1339_1340delA/wt	1
13	c.1286C>A(p.A429E)/wt	1
14	c.1336C>T(p.Q446X)/wt	1
15	IVS18+1G>A/wt	1
16	c.1673A>T(p.N558I)/wt	1
17	c.917insG/wt	1
18	c.946G>T(p.G316X)/wt	1
19	c.1687_1692insA/wt	1
20	wt/wt	18

wt: wild-type.

### Genetic analysis of *KCNJ10* among NSEVA patients lacking mutations in one or both alleles of *SLC26A4*

Among the 131 NSEVA patients lacking mutations in one or both alleles of *SLC26A4*, we identified the heterozygous missense mutation c.812G>A in *KCNJ10* in two cases (Fig. S1), with a mutation rate of 1.53% (2/131); the *KCNJ10* c.1042C>T mutation was not found. In the two cases with the *KCNJ10* c.812G>A mutation, Patient 195 carried no mutations in *SLC26A4* and Patient 1606 carried a heterozygous mutation in *SLC26A4* (c.225C>G). In addition, we screened *SLC26A4* and *KCNJ10* genes in the parents of Patient 195 (Table 3). Both Patient 195 and his father carried *KCNJ10* c.812G>A and wild-type *SLC26A4*; however, only Patient 195 developed NSEVA symptoms.

**Table 3:** The KCNJ10 c.812G>A mutation identified in Chinese patients with nonsyndromic enlargement of vestibular aqueduct (NSEVA).

Pedigree No.	Phenotype	*SLC26A4* mutation	*KCNJ10* mutation
195 probandPM	NSEVANormalNormal	wt/wtwt/wtwt/wt	c.812G>A (p.R271H)/wtc.812G>A (p.R271H)/wtwt/wt
1606 probandPM	NSEVANormalNormal	c.225C>G(p.L75L)/wt unknownUnknownUnknown	c.812G>A(p.R271H)/wtUnknownUnknown
2471 probandPM	NSEVANormalNormal	IVS7-2 A>G/c.2027T>A(p.L676Q)c.2027T>A(p.L676Q)/wtIVS7-2 A>G/wt	c.812G>A (p.R271H)wtc.812G>A (p.R271H)/wtwt/wt

wt, wild-type; P, father of proband (paternal); M, mother of proband (maternal).

### Genetic analysis of *KCNJ10* among NSEVA patients with two mutations in *SLC26A4*

As a control, we conducted *KCNJ10* mutation screening in 563 patients with NSEVA who carried two mutations in *SLC26A4*. We found 11 cases with a heterozygous *KCNJ10* c.812G>A mutation (1.95%, 11/563) and three cases with a heterozygous *KCNJ10* c.1042C>T mutation (0.53%, 3/563) (Fig. S1). We performed further testing for *KCNJ10* in one of the pedigrees containing 11 subjects with the *KCNJ10* c.812G>A mutation. Our result showed that Patient 2471 shared *SLC26A4* c.2027 T>A and *KCNJ10* c.812 G>A with his father, and *SLC26A4* IVS7-2 A>G with his mother. Patient 2471, with compound heterozygous mutations in *SLC26A4*, developed NSEVA, whereas his father, with double heterozygosity in both *SLC26A4* and *KCNJ10*, was not affected (Table 3).

We examined three patients with NSEVA in Pedigrees 4769 and 4814 who had biallelic *SLC26A4* mutations and the *KCNJ10* c.1042C>T mutation (Table 4). Hearing examinations showed that the hearing capabilities of the parents were normal, and high-resolution temporal bone computed tomography (CT) scans showed that EVA was not present in the parents. Because both parents in Pedigree 1134 were deceased, neither hearing examinations nor DNA sequencing could be done. However, the proband from Pedigree 3 reported to us that his parents had no hearing problems. We sequenced the *KCNJ10* and *SLC26A4* genes from the family members in Pedigrees 4769 and 4814. For Pedigree 4769, the proband’s mother carried the *SLC26A4* c.2168A>G (p.H723R) and *KCNJ10* c.1042C>T (p.R348C) mutations. For Pedigree 4814, the proband’s father carried the monoallelic *SLC26A4* IVS7-2A>G and *KCNJ10* c.1042C>T (p.R348C) mutations. IVS7-2A>G and c.2168A>G (p.H723R) are pathogenic mutations supported by both functional and molecular epidemiological studies [21]–[23]. They are also prevalent *SLC26A4* hotspot mutations in Chinese NSEVA. The finding that normal-hearing individuals carried both the *KCNJ10* c.1042C>T mutation and a *SLC26A4* pathogenic mutation suggests that *KCNJ10* c.1042C>T may not always act together with *SLC26A4* mutation in digenic inheritance related to the etiology of NSEVA in Chinese subjects.

**Table 4:** Identification of the *KCNJ10* c.1042C>T mutation in three NSEVA pedigrees with biallelic mutations in *SLC26A4*.

Pedigree No.	Phenotype	*SLC26A4* mutation	*KCNJ10* mutation
4769 probandPM	NSEVANormalNormal	IVS7-2 A>G/c.2168 A>G (p.H723R)IVS7-2 A>G/wtc.2168 A>G (p.H723R)/wt	c.1042 C>T(p.R348C)/wtwt/wtc.1042 C>T(p.R348C)/wt
4814 probandPMS	NSEVANormalNormalNSEVA	IVS7-2 A>G/IVS7-2 A>GIVS7-2 A>G/wtIVS7-2 A>G/wtIVS7-2 A>G/IVS7-2 A>G	c.1042 C>T(p.R348C)/wtc.1042 C>T(p.R348C)/wtwt/wtc.1042 C>T(p.R348C)/wt
1134 probandPM	NSEVANormalNormal	IVS7-2 A>G/c.249 G>A (p.W83X)UnknownUnknown	c.1042 C>T(p.R348C)/wtUnknownUnknown

wt, wild-type; P, father of proband (paternal); M, mother of proband (maternal); S, sibling of proband.

### Genetic analysis of *KCNJ10* among subjects with inner ear malformation

Among 48 patients with NSEVA and non-EVA inner ear malformations, we identified one case (1/48, 2.1%) with the missense mutation c.812G>A in *KCNJ10*.

### Genetic analysis of *KCNJ10* among subjects with conductive hearing loss

Among the 96 inpatients with conductive hearing loss, we detected *KCNJ10* heterozygous c.812G>A in four cases (4/96, 4.2%), heterozygous c.811C>T in one case (1/96, 1.0%), and heterozygous c.1042C>T in one case (1/96, 1.0%).

### Genetic analysis of *KCNJ10* among normal-hearing subjects

We completed *KCNJ10* mutation screening in 133 normal-hearing control subjects with no family history of hereditary hearing loss. Seven subjects carried a single heterozygous mutation of c.812G>A in *KCNJ10* (7/133, 5.3%), and one subject carried a single heterozygous mutation of c.1042C>T in *KCNJ10* (1/133, 0.8%). No other mutations in *KCNJ10* were detected. No statistical difference in the *KCNJ10* c.812G>A and c.1042C>T mutations was observed between the normal-hearing group and the NSEVA cases with zero, one, or two mutations in *SLC26A4*, patients with inner ear malformation, or patients with conductive hearing loss (χ^2^ *= 6.287, P = 0.179*).

## Discussion

NSEVA/PS is an autosomal recessive disorder that results in sensorineural hearing loss and is associated with mutations in the *SLC26A4* gene. The exact causal relationship between the *SLC26A4* genotype and pathological phenotypes remains controversial [24]. Some patients with NSEVA have biallelic mutations in *SLC26A4*, including homozygous mutations and compound heterozygous mutations, whereas others lack mutations in one or both alleles of *SLC26A4*. In addition, the ratios of *SLC26A4* biallelic and monoallelic mutations vary across different populations. For example, double and single mutation rates are 81% and 11% in South Korea [13], 77% and 13% in France [25], 47% and 13% in Japan [26], and 88% and 10% in China, respectively [27]. Several reasons may explain this seemingly confusing phenomenon. First, the efficiency and effectiveness of mutation screening are limited. Mutations in the promoter and intronic cryptic splicing regions could be missed [28]. Benign *SLC26A4* polymorphic variants could be misrecognized as pathogenic alleles [24]. Furthermore, the pathogenesis of NSEVA/PS, as for many complex multigenic disorders, may result from interactions among multiple genes and environmental factors. In patients with NSEVA/PS, mutations in *FOXI1*, a transcription factor of *SLC26A4*, have been identified either alone or in combination with mutations in *SLC26A4* [28]. Single mutations in both *SLC26A4* and *KCNJ10* have been suggested as another potential etiological mechanism [14].

Yang et al. [14] proposed that single mutations in both *SLC26A4* and *KCNJ10*, an inward rectifier potassium channel, lead to digenic NSEVA. *KCNJ10* c.1042C>T was shown to reduce K^+^ conductance activity and was therefore considered a potential pathological mutation. *KCNJ10* c.1042C>T was not found in ESP 6500 and was regarded as a pathogenic SNV by 1000 Genomes. However, in our study, *KCNJ10* c.1042C>T was not found in patients with NSEVA with zero or one *SLC26A4* mutation (n = 131). But the facts that *KCNJ10* c.1042C>T was found in a normal-hearing control subject and that normal hearing parents of patients with NSEVA with two *SLC26A4* mutations carried both *KCNJ10* c.1042C>T and *SLC26A4* pathogenic mutations suggest that *KCNJ10* c.1042C>T might be a benign variant in the Chinese population.

The most frequently identified mutation of *KCNJ10* in this study was c.812G>A (p.R271H). *KCNJ10* c.812G>A was not found in ESP 6500 and was regarded as a benign SNV by 1000 Genomes. It was found in patients with NSEVA who carried no mutation, a monoallelic mutation, or two mutations in *SLC26A4*; in patients with inner ear malformation; in patients with conductive hearing loss; and in normal-hearing individuals. The occurrence rate of c.812G>A had no significantly difference among the above five group people, suggest that *KCNJ10* c.812G>A is a benign variant in the Chinese population. Interestingly, the hearing in the two individuals with the *SLC26A4* mutations and *KCNJ10* c.1042C>T (p.R348C) mutations was normal. It may be due to the likelihood of incomplete penetrance of the p.R348C mutation influenced by additional environmental factors, genetic modifiers or difference in genetic/ethnical background. Our observations also suggested another possibility that *KCNJ10* c.1042C>T (p.R348C) is a benign variant in the Chinese population.

Similar to our study, other genetic studies in patients with EVA/PS have failed to find convincing evidence that *KCNJ10* mutations contribute to these phenotypes. Jonard et al. [29] screened 25 patients with unilateral deafness and unilateral EVA, but found no mutations in *KCNJ10*. Mercer et al. [30] screened 51 patients with EVA and found no mutations in *KCNJ10*. Chen et al. [31] screened *SLC26A4* and *KCNJ10* in patients with bilateral deafness and inner ear malformations and found no mutations in *KCNJ10* in the 15 patients who had one or no *SLC26A4* mutations. Landa et al. screened *KCNJ10/FOXI1* in 68 EVA or Pendred syndrome patients with with monoallelic mutations of SLC26A4. They found no evidence for an association between mutations of *KCNJ10* or *FOXI1* with SLC26A4 mutations in the pathogenesis of EVA or Pendred syndrome [32].

To our knowledge, this report is the largest study to screen *KCNJ1*0 in patients with NSEVA with two, one, or no *SLC26A4* mutations. In addition, we screened *KCNJ1*0 in patients diagnosed with conductive hearing loss having non-EVA inner ear malformations. Interestingly, no statistical differences in *KCNJ10* mutation rates were detected among all of the above groups and the normal-hearing control group.

## Conclusions

*SLC26A4* is the major genetic cause in Chinese NSEVA patients, accounting for 87.59%. *KCNJ10* may not be a contributor to NSEVA. Other genetic or environmental factors are possibly playing a role in the etiology of Chinese NSEVA patients with zero or monoallelic *SLC26A4* mutation.

## Supporting Information

Figure S1 **The representative chromatograms of the Sanger sequencing data for wild, c.812G>A and c.1042C>T of KCNJ10. A: wild type of KCNJ10 in the 812 locus.** B: sense strand of 812G>A mutation: wild type of KCNJ10 in the 1042 locus. D: sense strand of 1042C>T mutation. E: antisense strand of 1042C>T mutation.
(TIF)
