# Supplementary figures and images for: KCNJ10 May Not Be a Contributor to Nonsyndromic Enlargement of Vestibular Aqueduct (NSEVA) in Chinese Subjects
Source: PLoS One. 2014 Nov 5;9(11):e108134. doi: 10.1371/journal.pone.0108134 (PMC4220913; doi:10.1371/journal.pone.0108134)

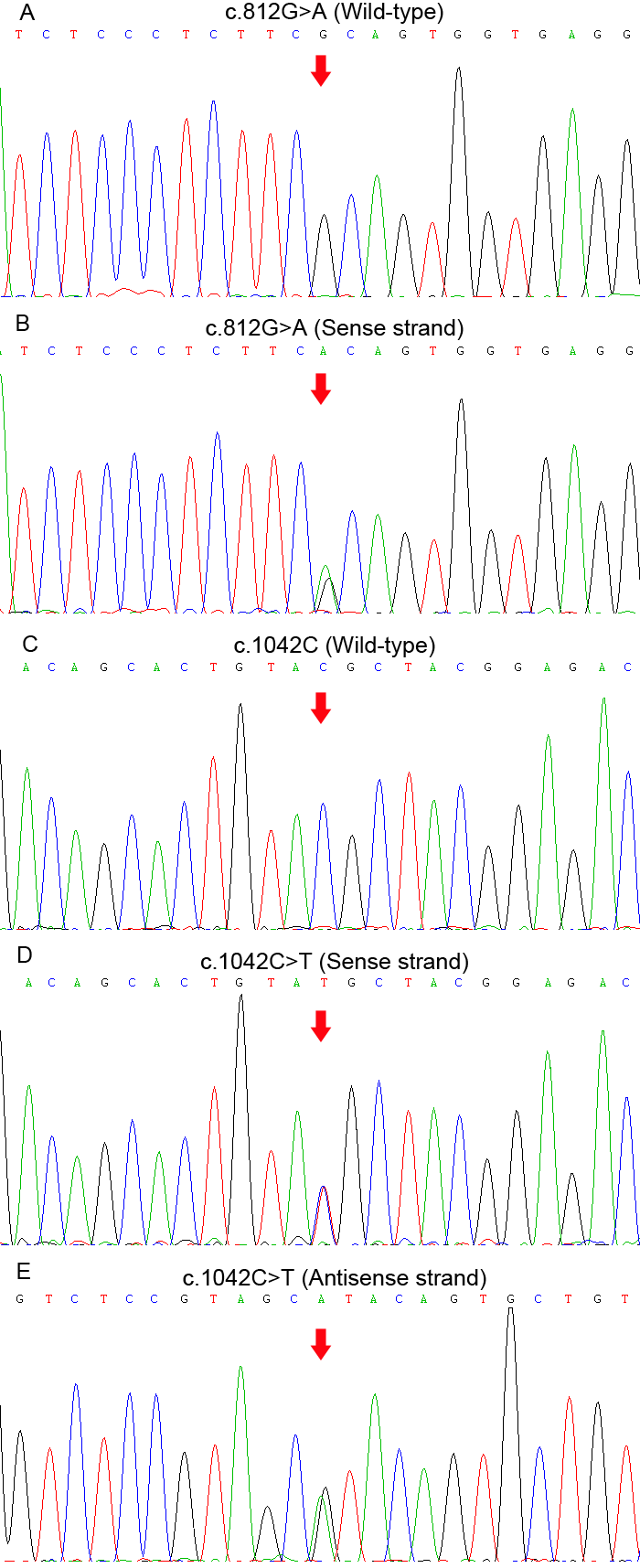

Supplement: Figure S1 — The representative chromatograms of the Sanger sequencing data for wild, c.812G>A and c.1042C>T of KCNJ10. A: wild type of KCNJ10 in the 812 locus. B: sense strand of 812G>A mutation: wild type of KCNJ10 in the 1042 locus. D: sense strand of 1042C>T mutation. E: antisense strand of 1042C>T mutation. (TIF) [file pone.0108134.s001.tif]
